# Control of the Mdm2-p53 signal loop by β-arrestin 2: The ins and outs

**DOI:** 10.18632/oncotarget.28065

**Published:** 2021-12-21

**Authors:** Elodie Blondel-Tepaz, Hervé Enslen, Mark G.H. Scott

**Keywords:** β-arrestin, Mdm2, p53, RanGAP1, SUMO

***Comment on:** The RanBP2/RanGAP1-SUMO complex gates β-arrestin2 nuclear entry to regulate the Mdm2-p53 signaling axis by Blondel-Tepaz et al. Oncogene. 2021; 40:2243–57. https://doi.org/10.1038/s41388-021-01704-w. [PubMed]*

Mdm2 is a major cellular inhibitor of p53. Small molecules designed to block the Mdm2-p53 interaction have been developed as an approach for the treatment of cancer with wild-type p53 [1]. In light of this therapeutic interest continued study of mechanisms that control the Mdm2-p53 signal loop is therefore of central importance.

**Figure 1:**
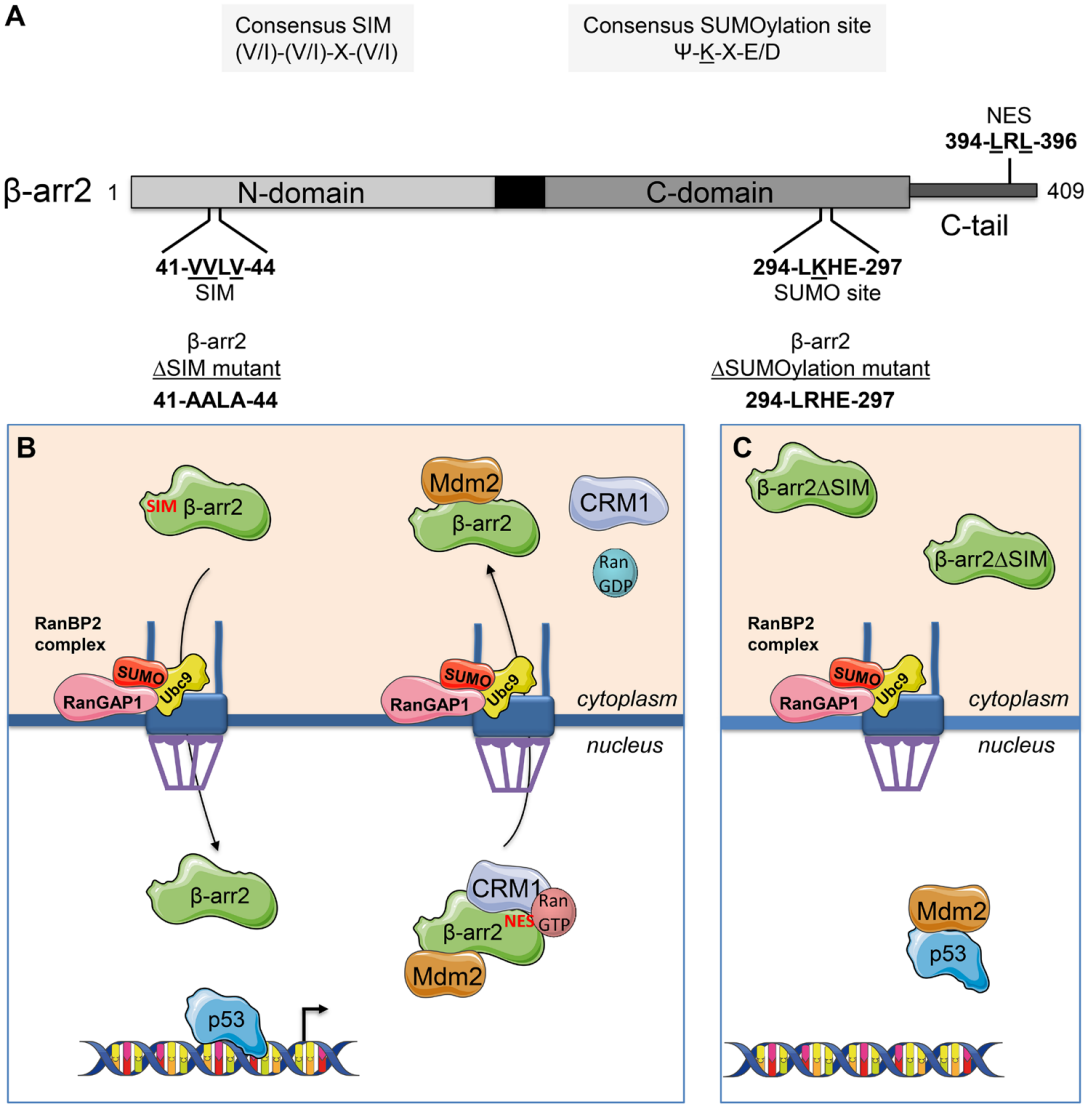
Model outlining the nucleocytoplasmic function of β-arr 2. (**A**) Schematic diagram indicating the SIM, SUMOylation site and NES in β-arr 2, and the ∆SIM and ∆SUMOylation site mutants used in the study. (**B**) Nucleocytoplasmic shuttling function of β-arr 2 with active import and export events results in displacement of Mdm2. (**C**) Defective nuclear import with the β-arr 2∆SIM mutant results in loss of Mdm2 displacement.

The β-arrestins (β-arrs) are two scaffold proteins initially appreciated for their roles in the desensitization and endocytosis of G protein-coupled receptors [2, 3]. They also dynamically regulate the activity and/or subcellular distribution of key intracellular signalling partners including Mdm2 [4–6]. Despite strong sequence homology, β-arr 1 and β-arr 2 present differential subcellular distributions. While β-arr 1 is found distributed both in the nucleus and cytoplasm, β-arr 2 displays an apparent cytoplasmic localization. This is due to constitutive ejection of β-arr 2 from the nucleus through a leptomycin B-sensitive pathway, directed via a nuclear export signal (NES) harboured by β-arr 2 (Figure 1A) that is absent in β-arr 1 [7, 8]. In addition, β-arr 2 is actively imported into the nucleus indicating that it undergoes continual nucleocytoplasmic trafficking. This shuttle function of β-arr 2 results in the displacement of Mdm2 from the nucleus to the cytoplasm, with an associated increase in p53 signalling and cell cycle arrest [5, 6].

Contrasting with the well characterized nuclear export mechanism of β-arr 2, knowledge on its entry mechanism(s) into the nucleus and functional impact on Mdm2-p53 signalling remains incomplete. SUMOylation is a post-translational modification that regulates the activity and localization of protein targets including nuclear targeting. β-arr 2 can be SUMOylated [9–11], but no information was available on how small ubiquitin-like modifier (SUMO) might regulate β-arr 2 nucleocytoplasmic shuttling. We therefore explored if SUMO could participate in controlling β-arr 2 nucleocytoplasmic shuttling function. In addition to SUMOylation sites for covalent conjugation of SUMO on a lysine residue, SUMO interaction motifs (SIMs) composed of a short stretch of hydrophobic residues can mediate non-covalent interaction with SUMO resulting in targeting of SIM-containing proteins to SUMOylated protein partners [12, 13]. Using a variety of *in vitro*, *in silico* and cell-based approaches we characterized both a SUMOylation site and SIM in β-arr 2 [14] (Figure 1A). Fusion of SUMO to β-arr 2 was recently found to increase its targeting to the nuclear rim [11]. We found, however, that SUMOylation was not required for nuclear import but that the SIM contained in β-arr 2 was [14]. We also found that the β-arr 2 SIM promotes association with the multimolecular RanBP2/RanGAP1-SUMO nucleocytoplasmic transport hub that resides on the cytoplasmic filaments of the nuclear pore complex. RanBP2 has been shown to act as a platform for nuclear import of a subset of import cargos [15]. We therefore tested the effect of depletion of the RanBP2/RanGAP1-SUMO complex on β-arr 2 nuclear import and indeed found it to be required, indicating its functional importance in β-arr 2 cytonuclear trafficking. RanBP2 has been proposed to enhance nuclear import by at least two mechanisms. Firstly, import receptor-independent interaction of selected cargos with RanBP2 can increase efficiency of nuclear import [15]. Secondly, it serves as a binding site for importin β1 retaining the transport receptor in association with the nuclear pore complex and reducing the active concentration of import receptors required for efficient transport [16, 17]. Interestingly, in this context, a recent study identified a nuclear localization signal in β-arr 2 and importin β1-dependent nuclear import [18] indicating that β-arr 2 nuclear import probably involves multiple steps coordinated by RanBP2. In summary, our findings demonstrate that the β-arr 2 SIM targets it to the RanBP2/RanGAP1-SUMO complex, which gates β-arr 2 nuclear entry (Figure 1B).

We next analyzed the function of the β-arr 2 SIM on the downstream Mdm2-p53 signal loop. Due to the defective nuclear import of a β-arr 2∆SIM mutant it lost the capacity to titrate Mdm2 from the nucleus to the cytoplasm observed with wild-type β-arr 2 (Figure 1B and 1C). Using non-small cell lung carcinoma and breast tumour cell lines we also found the enhancing effect of β-arr 2 on p53 signalling was lost with the β-arr 2∆SIM mutant. The ∆SIM mutant therefore gives rise to the same defective p53 signalling effect as a β-arr 2∆NES mutant, which also fails to displace Mdm2 from the nucleus. Our study [14] uncovering the role of a β-arr 2 SIM nuclear entry checkpoint, coupled with its active nuclear export provide an emerging picture of regulatory points that influence β-arr 2-mediated regulation of the Mdm2-p53 axis (Figure 1B). Further studies will be required to determine the full role of the SIM in β-arr 2 compartmentalization and if β-arr 2 cytonuclear function is disrupted in cancer settings.
